# Dietary diversity and practice of pregnant and lactating women in Ethiopia: A systematic review and meta‐analysis

**DOI:** 10.1002/fsn3.2228

**Published:** 2021-03-16

**Authors:** Zebenay Workneh Bitew, Ayinalem Alemu, Ermias Getaneh Ayele, Teshager Worku

**Affiliations:** ^1^ Department of Pediatric Nursing School of Nursing St. Paul’s Hospital Millennium Medical College Addis Ababa Ethiopia; ^2^ Ethiopian Public Health Institute Addis Ababa Ethiopia; ^3^ College of Health and Medical Sciences School of Nursing and Midwifery Haramaya University Harar Ethiopia

**Keywords:** Ethiopia, low dietary diversity, minimum dietary diversity, undernutrition

## Abstract

The dietary diversity of pregnant and lactating women remains unacceptably poor in resource‐limited countries such as Ethiopia. Despite the presence of inconsistent and inconclusive small‐scale studies, it is difficult to portray an actual picture of dietary diversity and dietary practices of women in Ethiopia. This study aimed to identify the prevalence of dietary diversity, dietary practice, and dietary patterns of pregnant and lactating women in Ethiopia. Electronic and gray literature sources were explored. A total of 3,256 articles were found, of which 38 were included in the final analysis. The data were analyzed by using STATA version 15. The pooled estimates were presented using random‐effects models due to considerable heterogeneities among studies. In this study, 16,412 pregnant and lactating women were included. The pooled prevalence of low, medium, and high dietary diversity scores of pregnant women was 37.1%, 41.55%, and 39.3%, respectively. Likewise, low, medium, and high dietary diversity scores of lactating women were 50.31%, 41.22%, and 9.1%, respectively. The mean dietary diversity of pregnant and lactating women was 3.99 ± 0.20. Regarding the minimum dietary diversity, 56.6% of pregnant women and 50.21% of lactating women were found to have inadequate dietary diversities. Two‐third (65.7%) of pregnant women were found to have poor dietary practice. Starchy foods were the main staple foods of study subjects, whereas organ meats were least consumed food types. The dietary diversity score, minimum dietary diversity, and dietary practices of women are suboptimal and below WHO and FAO recommendations. This could lead to both macro‐ and micronutrient deficiencies. Policymakers, program managers, healthcare workers, and stakeholders need to redesign nutrition promotion and intervention programs to alleviate this issue.

## Graphical abstract

The abstract is about the pooled prevalence of dietary diversity and practice of pregnant and lactating women in Ethiopia.

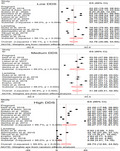

## 1 INTRODUCTION

Minimum dietary diversity of women (MDD‐W) is a dichotomous indicator of whether or not a woman (15–49 years) consumed the recommended food types in a specific period, usually in a 24‐hr period (FAO, 2016a). It is a proxy indicator of dietary quality and micronutrient status of women (Chakona & Shackleton, 2017; FAO, 2016a; Hoddinott & Yohannes, 2002). It also implied dietary adequacy (Rathnayake et al., 2012), and a well‐balanced diet is very crucial for pregnant and lactating women as they support two lives, the mother and their fetus or infants (Koletzko et al., 2019; Kominiarek & Rajan, 2016; Thorne‐Lyman et al., 2010). During pregnancy, women are recommended to have adequate dietary diversity that can assure adequate energy, macronutrient and micronutrient to support fertility, pregnancy, and positive birth outcomes and future health of the offspring (Caut et al., 2019; Ho et al., 2016). Lactating women are also in need of appropriate diet for their healthiest life and for growth and development of their infants (Kominiarek & Rajan, 2016).

Dietary inadequacy among pregnant and lactating women is not very uncommon in the globe (Marangoni et al., 2016). The burden is ominously higher among pregnant and lactating women in resource‐poor countries where women have monotonous dietary patterns and inadequate dietary diversity, which could lead to undernutrition (micronutrient or macronutrient undernutrition) (Haileslassie et al., 2013; Kang et al., 2019; Perumal et al., 2013; Sirotin et al., 2012). This is because the feeding practices of women in resource‐limited settings are nondiversified, starch‐based foods, nonanimal products, and limited consumption of vegetables and fruits (Arimond et al., 2010; Ruel, 2003). The issue is not different in Ethiopia, where a significant number of women have inadequate dietary diversity (Nguyen et al., 2013), leading to micronutrient deficiency among Ethiopian women (Haidar, 2010).

Undernutrition during pregnancy secondary to inadequate diversity can lead to obstetric and neonatal complications such as preterm birth, intrauterine growth retardation and low birthweight, and stillbirth (Black et al., 2013; Gernand et al., 2016; Kibret et al., 2019). Undernutrition of lactating women as a result of poor dietary habits and feeding practices could also lead to poor health and nutritional status of children (Tafese & Kebebu, 2017).

In Ethiopia, there are inconsistent and inconclusive evidences about the minimum dietary diversity (MDD) of pregnant and lactating women. The prevalence of inadequate MDD among pregnant women is more than 50% in most of the studies ranging as high as 74% (Aliwo et al., 2019; Desta et al., 2019; Gizahewu et al., 2019; Hailu & Woldemichael, 2019; Kumera et al., 2015; Yeneabat et al., 2019). There is a pronounced variation in the prevalence of inadequate MDD in lactating women ranging from 25% in Amhara region (Diddana, 2019) to 94.7% in Tigray region (Sitotaw et al., 2017). The dietary diversity scores (DDSs) of pregnant and lactating women in Ethiopia are also very inconsistent and the high prevalence of low DDS was reported in a study done in Oromia region, where 79% of lactating women had low DDS (Duko et al., 2018). The low DDS of pregnant women in Ethiopia was as low as 9.2% (Kobiro et al., 2020) and as high as 68.8% (Demilew et al., 2020). Similarly, the dietary practices (poor or good) of pregnant women were found to be highly variable. It was depicted in reviewed studies that most of the pregnant women had poor dietary practice (Alemayehu & Tesema, 2015; Demilew et al., 2020; Diddana, 2019; Nana & Zema, 2018; Tenaw et al., 2018; Tolera et al., 2018). In addition, the types of foods consumed by pregnant and lactating women were found to be very monotonous and starchy staples were the common food types consumed by Ethiopian women. The mean DDSs of pregnant and lactating were also very discrepant that ranged from 1.1 ± 0.55 in Tigray region (Sitotaw et al., 2017) to 7 ± 1.14 in Southern Nations, Nationalities, and Peoples (SNNP) regional state (Kobiro et al., 2020).

The aforementioned explanations imply that evidences are scant about suboptimal dietary practice of pregnant and lactating women in Ethiopia, which probes us to do this systematic review and meta‐analysis. The outputs of this study will be vital for policymakers, program planners, healthcare workers, and stakeholders who are working in this sensitive issue.

Therefore, this systematic review and meta‐analysis was aimed to compute the pooled prevalence of minimum dietary diversity, dietary diversity scores, mean dietary diversity scores, feeding practices, and meal frequencies of pregnant and lactating women in Ethiopia. The food types consumed by pregnant and lactating women in Ethiopia were also analyzed.

**Key messages**

- The dietary practice of both pregnant and lactating women in Ethiopia is poor.
- The dietary diversity and practices of pregnant women in Ethiopia are below WHO and FAO recommendations.
- Suboptimal dietary diversity and practices among pregnant and lactating women could be associated with nutritional deficiencies, mainly micronutrient deficiencies.

## 2 METHODS

### 2.1 Literature searching strategies

The preferred reporting items for systematic review and meta‐analysis (PRISMA) (Moher et al., 2009) were followed in the write‐up process of this systematic review and meta‐analysis. The studies were retrieved comprehensively from both electronic databases and gray literature sources. The reputable databases including PubMed, MEDLINE (EBSCOhost), EMBASE (Elsevier), CINAHL (EBSCOhost), Web of Science, Scopus, Science Direct and Food Science and Technology Abstracts (FSTA), and gray literature sources such as Google Scholar, Mednar, WorldCat, and Google were explored. In addition, the reference lists of all included studies were cross‐checked and searched accordingly. Searching was conducted by three authors (ZWB, EGA, and TW), independently. The fourth author (AA) played an indispensable role in the compilation process of the studies obtained during the searching process. While searching, the following key terms were used: (a) population (pregnant women, lactating women, breastfeeding women); (b) exposure (feeding practice); (c) outcome (dietary diversity score, DDS, minimum dietary diversity, low dietary diversity, MDD, inadequate/adequate dietary diversity, poor feeding practice, good feeding practice); (d) study design (cohort, cross‐sectional, prevalence, epidemiology, observational, descriptive); (e) study settings (community‐based or clinical‐based); and (f) location (Ethiopia, regions of Ethiopia). The Boolean operators such as “OR” and “AND” were used during the searching process. The key terms were checked for their appropriateness before the actual date of searching. During the searching process, only studies conducted in the English language were included. The EndNote X8 Reference Manager was used to manage literatures. In this study, data searching was conducted from inception to April 2020 and articles conducted from 2013 to April 2020 were included in the final analysis. Example of search strategy in PubMed is as follows: ((((((((((dietary diversity) OR minimum dietary diversity) OR MDD) OR dietary diversity scores) OR DDS) OR "Malnutrition"[Mesh]) OR under nutrition) AND "Pregnant Women"[Mesh]) OR lactating women) OR breastfeeding women) AND "Ethiopia"[Mesh].

### 2.2 Data extraction process

Data extraction was done by three authors (ZWB, EGA, and TW) independently after reviewing the abstracts and full texts of all included studies. In addition, the methodological qualities of the included studies were assessed by authors (ZWB, AA, and TW) independently. We contacted the corresponding authors for those studies we were unable to access the full texts. Finally, incomplete studies were excluded after failing to communicate with the corresponding authors.

### 2.3 Data abstraction and critical appraisal of the studies

Three authors (ZWB, EGA, and TW) did data extraction using structured and pretested data extraction checklist. The terms that were included in the extraction checklist were as follows: name and publication year, study region, study population, sample size, DDS, DDS + *SD*, MDD, dietary practice, meal frequency within 24 hr, and food groups. The fourth author (AA) actively involved in resolving disagreements arose between the three authors during the extraction process. Joanna Briggs Institute (JBI) checklists of cross‐sectional and cohort studies (Munn et al., 2015) were used to assess the qualities of the included studies for this systematic review and meta‐analysis. Critical appraisal was done by the two authors (ZWB and AA). The tools have yes, no, not applicable, and unknown options. 1 was given for yes and 0 for other options. Then, the scores were summed up and changed to percentages. The mean scores of the two reviewers were used for final decision of inclusion. Those studies with >50% were included in this systematic review and meta‐analysis (Figure S1). The author (TW) settled disagreements of the two authors in the critical appraisal process. The presence of publication bias for high heterogeneity was checked, and the asymmetry of the funnel plot and/or statistical significance of Egger's regression test (*p* <.05) (Sterne & Egger, 2001) were considered as presence publication bias.

### 2.4 Operationalization of the outcomes

The main outcomes in this study were DDS, MDD, and dietary practice among pregnant and lactating women in Ethiopia. The 10 food groups (grains, white roots and tubers, and plantains (starches); pulses (beans, peas, and lentils); nuts and seeds; dairy; meat, poultry, and fish; eggs; dark green leafy vegetables; other vitamin A‐rich fruits and vegetables; and other vegetables and other fruits) that were recommended by FAO (FAO, 2016b) for pregnant and lactating women were used to compute the outcome measures. Dietary diversity scores (DDSs) were classified into low, medium, and high. These were computed by summing up the total number of food items that were consumed per 24 hr. In most of the included studies, women with DDS below the first quartile were low DDS, whereas women with DDS below 2nd quartile and higher than the third quartile were classified under medium and high DDS, respectively. Similarly, some of the included studies classified DDS as low (≤3 food types), medium (4–6 food types), and high (≥6 food types). The minimum dietary diversity of the studies was classified as inadequate if the study subjects consume less than five food types per day and adequate MDD for those who consume five and above food types within a day. Dietary practices of study subjects were also classified as poor or good. The magnitudes of each outcome variable were computed by dividing the number of study subjects in each outcome measure to the total sample and then multiplying by 100. The other outcomes were food types and minimum meal frequency, and they were calculated in the same fashion. The binomial distribution formula was used to compute the standard errors for each study.

### 2.5 Data analysis and assessment of certainty in the findings

We extracted the data using the extraction format prepared in Microsoft Excel 2016 (Table 1). Then, the data were imported into STATA version 15 (STATA Corporation, College Station Texas) software for analysis of the pooled estimates of low DDS, medium DDS, high DDS, adequate MDD, inadequate MDD, poor dietary practice, and good dietary practice of pregnant and lactating women in Ethiopia. The pooled estimates were presented using forest plots, summary tables, and graphs. The pooled estimates of outcomes were reported with 95% CI. Heterogeneity among studies was explored by using forest plot and *I* ^2^ test and Cochrane Q statistics (Rücker et al., 2008). The *I* ^2^ values of 25%, 50%, and 75% were interpreted as low, medium, and high heterogeneity, respectively. For this review, *I* ^2^ ≥ 50% with *p*‐value of <.05 was declared as the presence of heterogeneity and justified. The statistical tests pinpointed that there was heterogeneity (Higgins & Thompson, 2002) among all studies included to compute MDD, DDS, dietary practice, and mean DDS among pregnant and lactating women in Ethiopia (*I* ^2^ > 95%, *p* <.001). To identify the possible sources of heterogeneity, sensitivity and subgroup analyses were conducted. In addition, analyses were done using both random‐effects and fixed‐effects models interchangeably. However, no significant differences were observed using the DerSimonian and Laird's random‐effects model (Borenstein et al., 2010; DerSimonian & Laird, 1986). In sensitivity analysis, the trim‐and‐fill analyses were done and justified accordingly. Subgroup analyses were done for MDD and DDS of pregnant and lactating women in Ethiopia. Funnel plots were drawn using the logs of event rates (MDD, DDS, and dietary practices) and the logs of standard errors of event rates. The possibility of publication biases was objectively examined using Egger's weighted correlation and Begg's regression tests (Begg & Mazumdar, 1994). Hence, the pooled estimates were determined using Duval and Tweedie's trim‐and‐fill analysis in the random‐effects model. This is done to minimize the random variations between the point estimates of the included studies.

**TABLE 1:** Detailed description of included studies (*N* = 38)

Author, year	Study area	Study population	Sample size	DDS			Dietary Practice		MDD		DDS ± *SD*	MF		Quality score
				Low	Medium	High	Poor	Good	Inadequate	Adequate		<3X	>4X	
Delil et al., 2018	SNNP	Pregnant	314	76	168	70	—	—	—	—	3.6 ± 2.27	—	—	6
Desta et al., 2019	Oromia	Pregnant	315	—	—	—	—	—	235	80	3.48 ± 2.46	315	—	8
Jemal & Awol, 2019	Tigray	Pregnant	412	160	—	252	—	—	—	—	—	412	—	8
Kobiro et al., 2020	SNNP	Pregnant	303	28	146	129	—	—	—	—	7 ± 1.3	290	13	6
Demilew et al., 2020	Amhara	Pregnant	694	440	—	254	556	138	—	—	—	449	245	8
Diddana, 2019	Amhara	Pregnant	604	—	—	—	331	273	154	450	—	604	—	8
Shenka et al., 2018	Dire Dawa	Pregnant	380	—	—	—	—	—	163	217	—	—	—	6
Duko et al., 2018	Oromia	Lactating	484	382	99	3	—	—	—	—	—	200	284	8
Yeneabat et al., 2019	Amhara	Pregnant	759	—	—	—	—	—	421	378	3.68 ± 2.1	—	—	6
Boke & Geremew, 2018	SNNP	Lactating	410	—	—	—	—	—	214	196	4.5 ± 1.6	389	21	6
Weldehaweria et al., 2016	Tigray	Lactating	346	195	151	—	—	—	—	—	3.4	346	—	6
Ali, 2019	Somalia	Pregnant	37	—	—	—	—	—	—	—	—	—	—	6
Hailu & Woldemichael, 2019	Oromia	Pregnant	413	—	—	—	—	—	228	185	—	413	—	6
Nigatu et al., 2018	Gambela	Pregnant	322	—	—	—	—	—	—	—	6 + 1.58	322	—	6
Haileslassie et al., 2013	Tigray	Lactating	400	—	—	—	—	—	—	—	—	400	—	6
Asayehu et al., 2017	SNNP	Pregnant	159	—	—	—	—	—	—	—	3.7	—	—	6
Zerfu & Biadgilign, 2018	Oromia	Pregnant	374	—	—	—	—	—	—	—	3.57 ± 1.7	—	—	8
Desalegn et al., 2018	SNNP	Pregnant	153	—	—	—	—	—	—	—	—	149	4	6
Sonko, 2016	SNNP	Pregnant	236	—	—	—	—	—	—	—	—	217	19	6
Alemayehu et al., 2015	Oromia	Lactating	342	82	145	110	—	—	—	—	4.9 ± 1.9	305	33	8
Kumera et al., 2015	Amhara	Pregnant	364	230	—	134	—	—	230	134	3.35 ± 0.77	250	75	6
Tikuye et al., 2019	SNNP	Lactating	478	126	294	58	—	—	—	—	4.27 ± 1.19	175	303	8
Lebso et al., 2017	SNNP	Pregnant	504	237	200	67	—	—	—	—	—	—	—	6
Eramo, 2018	SNNP	Lactating	266	183	76	7	—	—	—	—	3.34 + 0.53	266	—	6
Endalifer et al., 2019	Tigray	Pregnant	306	—	—	—	—	—	—	—	—	—	—	8
Samuel et al., 2020	SNNP	Pregnant	423	119	219	85	—	—	—	—	—	—	—	6
Aliwo et al., 2019	Amhara	Pregnant	647	—		—	—	—	444	203	—	363	284	6
Gizahewu et al., 2019	SNNP	Pregnant	211	—	—	—	—	—	144	67	—	35	180	6
Alemayehu & Tesema, 2015	Amhara	Pregnant	574	—	—	—	344	230	—	—	3.7 ± 0.8	534	40	6
Sitotaw et al., 2017	Tigray	Lactating	456	—	—	—	—	—	432	24	1.1 ± 0.3	399	57	6
Workicho et al., 2019	Oromia	Pregnant	1,393	—	178	1,215	—	—	—	—	—	—	—	10*
Julla et al., 2018	SNNP	Lactating	421	200	214	7	—	—	—	—	—	—	—	8
Engidaw, et al., 2019	Amhara	Lactating	398	—	—	—	—	—	103	295	4.94 ± 0.76	228	170	8
Abriha et al., 2014	Tigray	Pregnant	619	142	271	206	—	—	—	—	—	493	126	6
Zerihun, et al. 2016	Oromia	Lactating	619	—	—	—	—	—	173	446	4 ± 1.5	—	—	6
Tenaw et al., 2018	AA	Pregnant	322	—	—	—	211	111	—	—	—	—	—	6
Tolera et al., 2018	Oromia	Pregnant	338	—	—	—	247	91	—	—	—	—	—	8
Nana & Zema, 2018	Amhara	Pregnant	616	—	—	—	374	242	—	—	—	—	—	8

Abbreviations: *, cohort study; DDS, dietary diversity score; MDD, minimum dietary diversity; MF, meal frequency; *SD*, standard deviation.

## 3 RESULTS

### 3.1 Study selection

**FIGURE 1:**
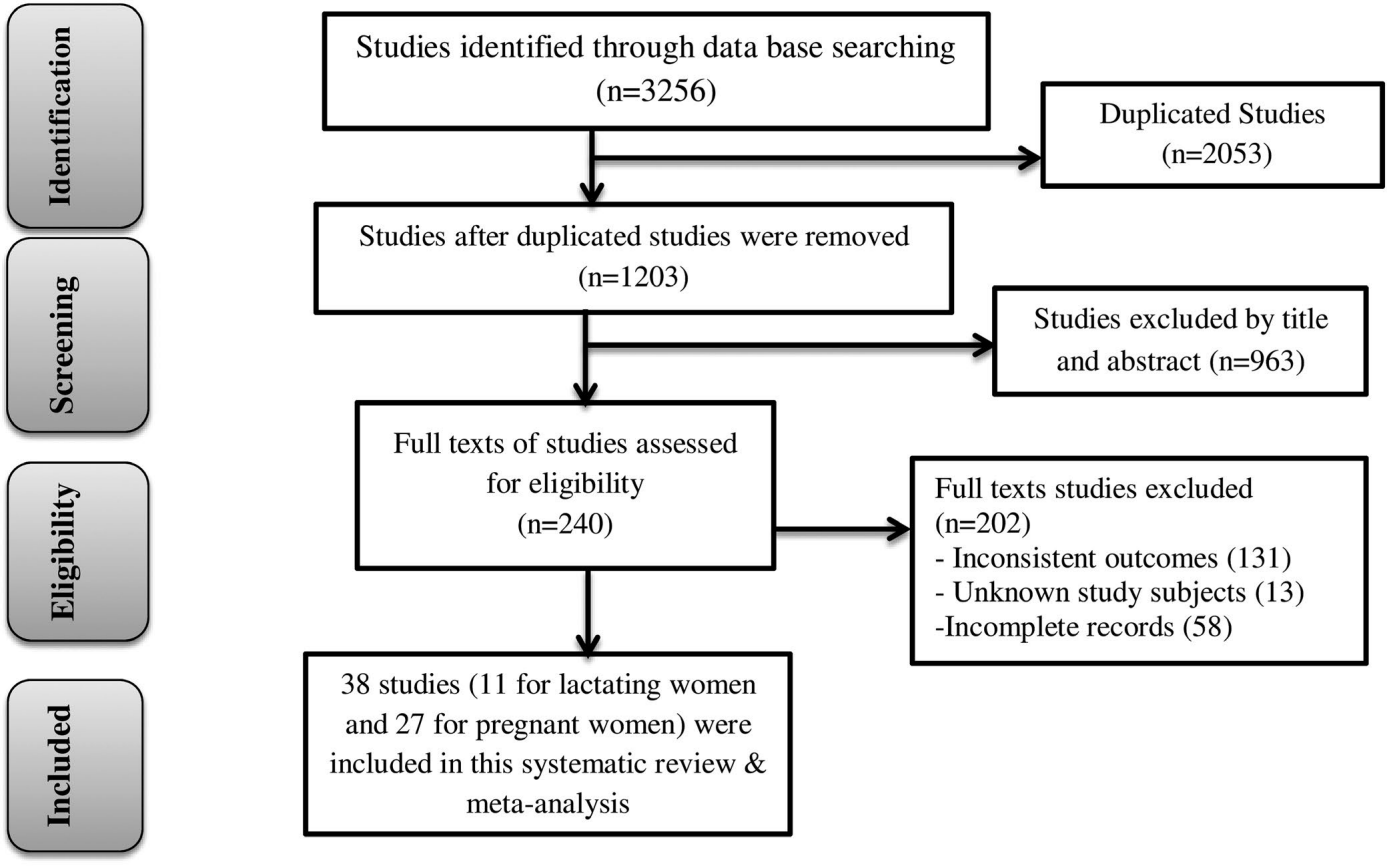
Flow diagram of studies included in this systematic review and meta‐analysis of DDS, MDD, and dietary practice in Ethiopia, 2020

During the initial search, 446 studies were found from both electronic database and nonelectronic gray literature sources. Of the total articles, 206 were duplicates and 189 were excluded after screening the titles and abstracts. The full texts of 51 articles were reviewed, and 13 articles were excluded due to inconsistent outcomes, unknown study subjects, and incompleteness of reports. Finally, 38 studies (Abriha et al., 2014; Alemayehu et al., 2015; Alemayehu & Tesema, 2015; Ali, 2019; Aliwo et al., 2019; Asayehu et al., 2017; Boke & Geremew, 2018; Kobiro et al., 2020; Delil et al., 2018; Demilew et al., 2020; Desalegn et al., 2018; Desta et al., 2019; Diddana, 2019; Duko et al., 2018; Endalifer et al., 2019; Engidaw et al., 2019; Eramo, 2018; Gizahewu et al., 2019; Haileslassie et al., 2013; Hailu & Woldemichael, 2019; Jemal & Awol, 2019; Julla et al., 2018; Kumera et al., 2015; Lebso et al., 2017; Nana & Zema, 2018; Nigatu et al., 2018; Samuel et al., 2020; Shenka et al., 2018; Sitotaw et al., 2017; Sonko, 2016; Tenaw et al., 2018; Tikuye et al., 2019; Tolera et al., 2018; Weldehaweria et al., 2016; Workicho et al., 2019; Yeneabat et al., 2019; Zerfu & Biadgilign, 2018; Zerihun et al., 2016) fulfilled the inclusion criteria and were included in this systematic review and meta‐analysis (Figure 1 and Table 1).

### 3.2 Characteristics of the studies

In this systematic review and meta‐analysis, 37 cross‐sectional studies and one cohort study were included. In the current study, a total of 16,412 study subjects were included of which 11,792 of them were pregnant women and 4,620 were lactating women. The sample size among pregnant women ranged from 37 (Ali, 2019) to 1,393 (Workicho et al., 2019), whereas it ranged from 266 (Eramo, 2018) to 619 (Zerihun et al., 2016) in the studies among lactating women. From the included studies, the majority (12 studies) (Asayehu et al., 2017; Boke & Geremew, 2018; Kobiro et al., 2020; Delil et al., 2018; Desalegn et al., 2018; Eramo, 2018; Gizahewu et al., 2019; Julla et al., 2018; Lebso et al., 2017; Samuel et al., 2020; Sonko, 2016; Tikuye et al., 2019) of them were from SNNP regional state. Eight studies were from Oromia region (Alemayehu et al., 2015; Desta et al., 2019; Duko et al., 2018; Hailu & Woldemichael, 2019; Tolera et al., 2018; Workicho et al., 2019; Zerfu & Biadgilign, 2018; Zerihun et al., 2016), and the other eight (Alemayehu & Tesema, 2015; Aliwo et al., 2019; Demilew et al., 2020; Diddana, 2019; Engidaw et al., 2019; Kumera et al., 2015; Nana & Zema, 2018; Yeneabat et al., 2019) were from Amhara region. The rest of the included studies were from Tigray region (Abriha et al., 2014; Endalifer et al., 2019; Haileslassie et al., 2013; Jemal & Awol, 2019; Sitotaw et al., 2017; Weldehaweria et al., 2016), Gambela region (Nigatu et al., 2018), Somalia region (Ali, 2019), Addis Ababa (Tenaw et al., 2018), and Dire Dawa (Shenka et al., 2018). Regarding the DDS of pregnant and lactating women, the highest proportion (79%) of low DDS was reported from a study done in Oromia region among lactating women (Duko et al., 2018). The highest proportion (94.7%) of inadequate DD was documented from a study conducted in Tigray region among lactating women (Sitotaw et al., 2017). From the included studies for computing the pooled prevalence of poor dietary practices of pregnant women in Ethiopia, the maximum proportion (80%) (Demilew et al., 2020) was reported from a study done in Amhara region. Coming to the food groups consumed by both pregnant lactating women in Ethiopia, almost all (95%) women ate starchy foods mainly cereals per 24 hr. Nuts and seeds were consumed by 6.6% of these study subjects, whereas organ meat is the least (1.8%) consumed (Table 2).

**TABLE 2:** Dietary pattern of pregnant and lactating women in Ethiopia

Author, year	Study population	Sample size	10 food groups										Others		
			Starch	Pulses	Nuts and seeds	Dairy	Meat	Eggs	Dark vegetables	VitA F. and Veg.	Other vegetables	Other fruits	Fats and oils	Legumes, nuts, and seeds	Organ meat
Delil et al., 2018	Pregnant	314	312	311	19	181	153	129	305	260	309	262	—	—	—
Desta et al., 2019	Pregnant	315	299	85	185	105	36	11	64	59	208	165	—	—	—
Jemal & Awol, 2019	Pregnant	412	406	213	221	203	131	141	348	168	336	92	—	—	—
Kobiro et al., 2020	Pregnant	303	298	300	16	122	61	303	260	210	288	240	—	—	—
Demilew et al., 2020	Pregnant	694	694	658	—	320	218	329	—	443	327	230	662	—	—
Diddana, 2019	Pregnant	604	604	598	69	154	158	176	280	145	312	290	—	—	—
Shenka et al., 2018	Pregnant	380	380	252		177	105	62	113	210	375	17	—	—	—
Duko et al., 2018	Lactating	484	484	—	—	74	19	29	106	79	391	391	—	160	2
Yeneabat et al., 2019	Pregnant	759	491	—	—	—	—	—	—	—	—	—	—	649	—
Boke & Geremew, 2018	Lactating	410	400	262	—	225	95	153	317	176	176	176	—	—	—
Weldehaweria et al., 2016	Lactating	346	344	—	—	43	59	63	65	85	145	145	123	239	—
Ali, 2019	Pregnant	37	36	—	—	35	31	21	18	22	17	28	35	—	27
Hailu & Woldemichael, 2019	Pregnant	413	367	310	140	215	34	149	227	182	—	—	248	—	—
Nigatu et al., 2018	Pregnant	322	322	—	—	81	158	30	197	187	286	56	310	160	1
Haileslassie et al., 2013	Lactating	400	369	297	—	22	15	20	158	102	347	94	366	—	9
Asayehu et al., 2017	Pregnant	159	153	69	——	25	5	7	—	—	156	10	128	—	—
Zerfu & Biadgilign, 2018	Pregnant	374	374	—	—	113	17	71	146	103	83	83	—	333	23
Desalegn et al., 2018	Pregnant	153	117	—	—	62	23	17	102	42	109	75	144	114	11
Sonko, 2016	Pregnant	236	235	38	—	186	37	93	—	215	—	165	220	—	—
Alemayehu et al., 2015	Lactating	342	323	303	—	99	34		229	154	267	120	291	—	15
Kumera et al., 2015	Pregnant	364	363	—	—	29	79	33	32	70	352	352	—	259	3
Tikuye et al., 2019	Lactating	478	477	—	—	129	29	38	437	225	374	374	—	249	29
Lebso et al., 2017	Pregnant	504	486	—	—	149	49	56	261	261	180	180	236	238	29
Eramo, 2018	Lactating	266	265	—	—	79	7	31	220	8	48	48	209	146	7
Endalifer et al., 2019	Pregnant	306	288	—	—	62	9	29	69	40	191	31	213	172	—
Samuel et al., 2020	Pregnant	423	423	—	—	281	133	75	295	86	244	244		283	24

Note

VitA F. and Veg.: other vitamin A‐rich fruits and vegetables.

### 3.3 Pooled prevalence of DDS and MDD among pregnant and lactating women in Ethiopia

**FIGURE 2:**
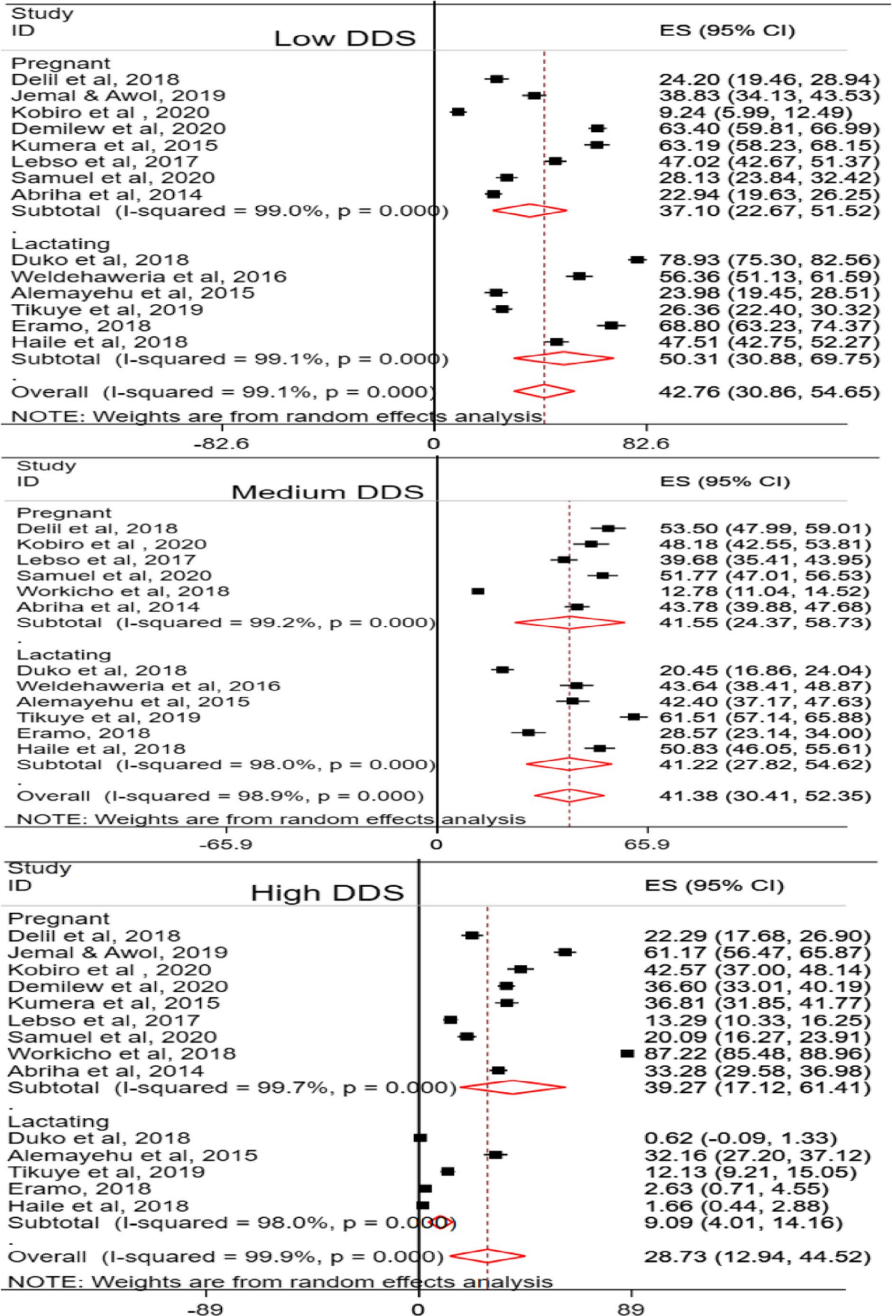
Dietary diversity scores (DDSs) of pregnant and lactating women in Ethiopia

The dietary diversity scores (DDSs) of pregnant and lactating women were classified into low, medium, and high. The pooled low DDS was computed from 15 studies, of which eight (Abriha et al., 2014; Kobiro et al., 2020; Delil et al., 2018; Demilew et al., 2020; Jemal & Awol, 2019; Kumera et al., 2015; Lebso et al., 2017; Samuel et al., 2020) of the studies were among pregnant women and six (Alemayehu et al., 2015; Duko et al., 2018; Eramo, 2018; Julla et al., 2018; Tikuye et al., 2019; Weldehaweria et al., 2016) were studies among lactating women. The pooled prevalence of low DDS among pregnant women in Ethiopia was found to be 37.1% (95% CI: 22.67, 51.52; *I* ^2^ = 99%, *p* ≤ .01). Likewise, the pooled prevalence of low DDS of lactating women was 50.31% (95% CI: 30.8, 69.75; *I* ^2^ = 99.1, *p* ≤ .01). The combined pooled prevalence of low DDS among pregnant and lactating women in Ethiopia was 42.76% (95% CI: 30.86, 54.65; *I* ^2^ = 99.1, *p* ≤ .01) (Figure 2). Similarly, the pooled prevalence of medium DDS was computed among six studies (Abriha et al., 2014; Kobiro et al., 2020; Delil et al., 2018; Lebso et al., 2017; Samuel et al., 2020; Workicho et al., 2019) on pregnant women and six studies (Alemayehu et al., 2015; Duko et al., 2018; Eramo, 2018; Julla et al., 2018; Tikuye et al., 2019; Weldehaweria et al., 2016) conducted on lactating women. In the present study, the pooled prevalence of medium DDS was 41.55% (95% CI: 24.37, 58.73; *I* ^2^ = 99.2%, *p* ≤ .01) among pregnant women and 41.22% (95% CI: 27.82, 54.62; *I* ^2^ = 98%, *p* ≤ .01) among lactating women in Ethiopia. The combined pooled prevalence of medium DDS of Ethiopian pregnant and lactating women was 41.38% (95% CI: 30.41, 52.35; *I* ^2^ = 98.9%, *p* ≤ .01) (Figure 2). In addition, the pooled prevalence of high DDS among pregnant and lactating women in Ethiopia was found to be 28.73% (95% CI: 12.94, 44.52; *I* ^2^ = 99.9%, *p* ≤ .01). The prevalence of high DDS among pregnant women was 39.27% (95% CI: 17.12, 61.41; *I* ^2^ = 99.7%, *p* ≤ .01), and this was computed using nine studies (Abriha et al., 2014; Kobiro et al., 2020; Delil et al., 2018; Demilew et al., 2020; Jemal & Awol, 2019; Kumera et al., 2015; Lebso et al., 2017; Samuel et al., 2020; Workicho et al., 2019). High DDS was also computed for lactating women using five studies (Alemayehu et al., 2015; Duko et al., 2018; Eramo, 2018; Julla et al., 2018; Tikuye et al., 2019), and it was found to be 9.1% (95% CI: 4.01, 14.16; *I* ^2^ = 98%, *p* ≤ .01) (Figure 2).

As it has been shown in the pooled estimates, there was a very high heterogeneity among the included studies since the *I* ^2^ statistic is greater than 95%. The possible sources of heterogeneity of low DDS, medium DDS, and high DDS were verified. Publication bias was primarily assessed using the funnel plot (See Figure S1) and objectively assessed using Egger's regression test. The *p*‐values of test results for low DDS, medium DDS, and high DDS were .285, .000, and .098, respectively. This revealed that there was a publication bias among studies used to compute the pooled prevalence of low DDS. The possible sources of heterogeneity were analyzed further through sensitivity analysis, specifically by using the trim‐and‐fill analysis. However, there was no any study considerably affecting the primary pooled estimates.

**FIGURE 3:**
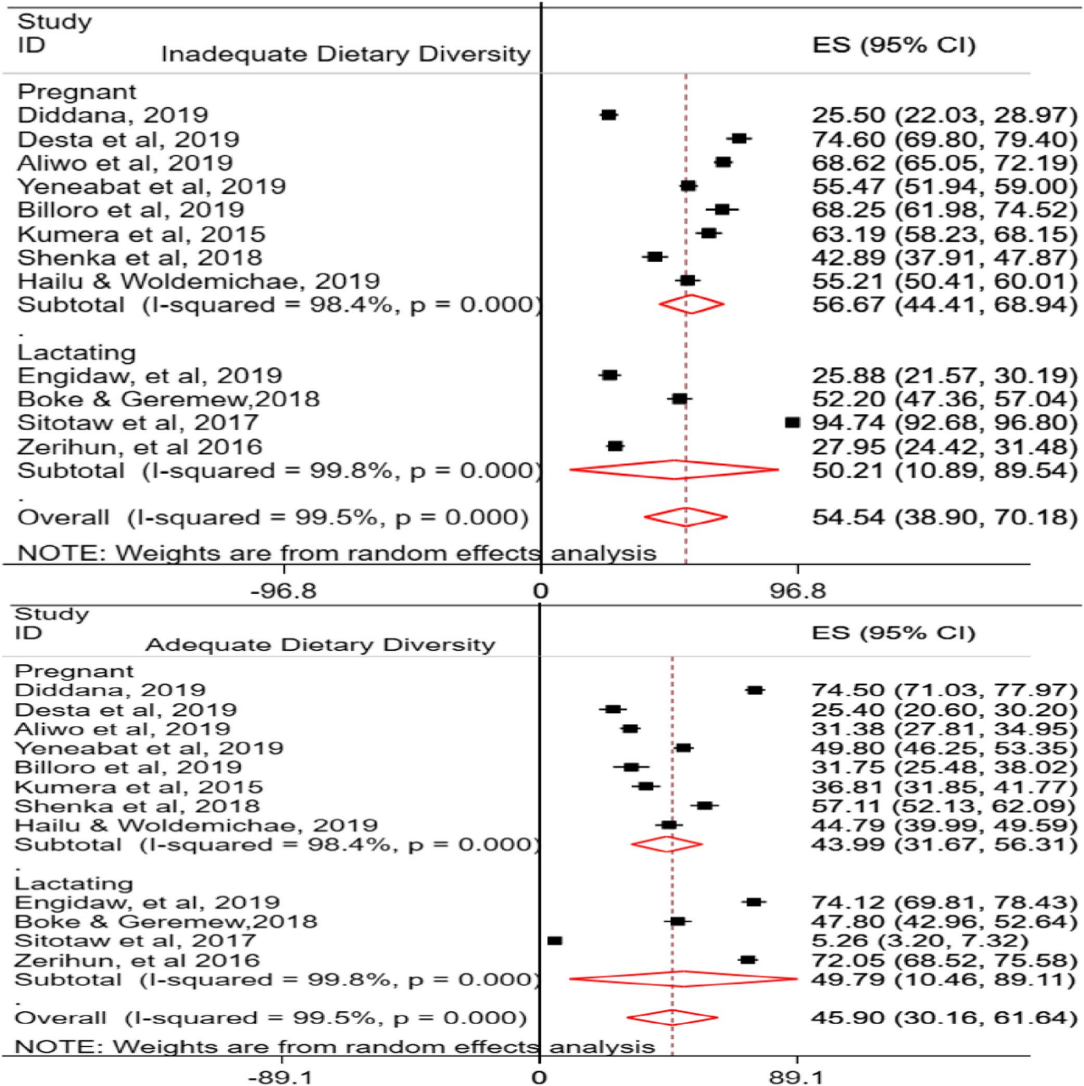
Dietary diversity of pregnant and lactating women in Ethiopia

**FIGURE 4:**
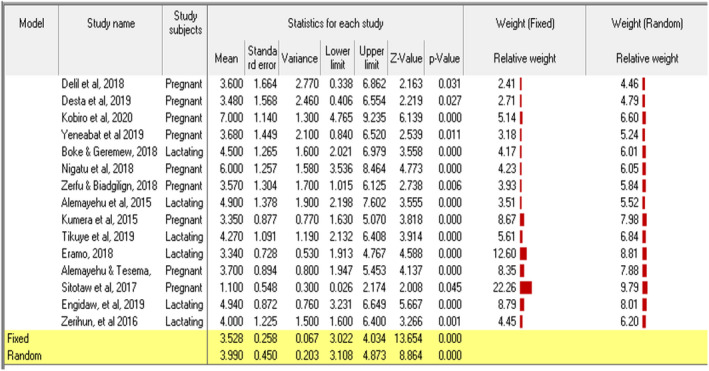
The mean DDS of pregnant and lactating women in Ethiopia

Regarding the minimum dietary diversity (MDD) of women in Ethiopia, 12 studies were used to compute the pooled prevalence, of which eight of the studies (Aliwo et al., 2019; Desta et al., 2019; Diddana, 2019; Gizahewu et al., 2019; Hailu & Woldemichael, 2019; Kumera et al., 2015; Shenka et al., 2018; Yeneabat et al., 2019) were for pregnant women and the rest (Boke & Geremew, 2018; Engidaw et al., 2019; Sitotaw et al., 2017; Zerihun et al., 2016) were studies conducted among lactating women. Inadequate MDD of pregnant women in Ethiopia was 56.67% (95% CI: 44.41, 68.94%; *I* ^2^ = 98.4%, *p* ≤ .01), whereas it was 50.21% (95% CI: 10.89, 89.54; *I* ^2^ = 99.8%, *p* ≤ .01) among lactating women. The pooled prevalence of inadequate MDD among pregnant and lactating women was 54.54% (95% CI: 38.9, 70.18; *I* ^2^ = 99.5%, *p* ≤ .01) (Figure 3). In this study, the pooled prevalence of adequate MDD among pregnant and lactating women in Ethiopia was 45.9% (95% CI: 30.16, 61.64; *I* ^2^ = 99.5%, *p* ≤ .01). Specifically, 44% of pregnant (95% CI: 31.67, 56.31; *I* ^2^ = 98.4, *p* ≤ .01) and 49.8% (95% CI: 10.46, 89.11; *I* ^2^ = 99.8%, *p* ≤ .01) of lactating women had adequate MDD (Figure 3). The possible sources of high heterogeneity were explored, and Egger's regression tests implied that there was no publication bias for both inadequate MDD (*p*‐value = .071) and adequate MDD (*p*‐value = .073). Sensitivity analysis was conducted to identify the possible sources, but no outlier study that significantly shifted the primary pooled estimates was found. In this study, the pooled mean DDS of pregnant and lactating women in Ethiopia was computed using 15 eligible studies (Alemayehu et al., 2015; Alemayehu & Tesema, 2015; Boke & Geremew, 2018; Kobiro et al., 2020; Delil et al., 2018; Desta et al., 2019; Engidaw et al., 2019; Eramo, 2018; Kumera et al., 2015; Nigatu et al., 2018; Sitotaw et al., 2017; Tikuye et al., 2019; Yeneabat et al., 2019; Zerfu & Biadgilign, 2018; Zerihun et al., 2016). Hence, the pooled mean DDS of pregnant and lactating women was found to be 3.99 ± 0.20, 95% CI: 3.11, 4.87, *I* ^2^ = 62.9%, *p* =.001 (Figure 4).

### 3.4 Subgroup analysis of DDS and MDD of pregnant and lactating women in Ethiopia

Regarding the DDS of pregnant and lactating women in Ethiopia, the highest pooled prevalence of low DDS was in Amhara region (63.3%, 95% CI: 57.54, 69). The highest prevalence of medium DDS and high DDS was in SNNP regional state (47.8%, 95% CI: 39.9, 55.6) and Tigray region (47.2%, 95% CI: 19.9, 74.5), respectively. Coming to the MDD of pregnant and lactating women in Ethiopia, more than two‐third (68.9%, 95% CI: 18.1, 119.7) of women in Tigray region and Dire Dawa (others) had inadequate MDD. Likewise, around one‐third (31.1%, 95% CI: −19.7, 82) of women had adequate MDD in Tigray and Dire Dawa (Table 3).

**TABLE 3:** Subgroup analysis of DDS and MDD among pregnant and lactating women in Ethiopia

Variables	# of studies	Low DDS (%) (95% CI)	# of studies	Medium DDS (%) (95% CI)	# of studies	High DDS (%) (95% CI)
*Study regions*						
Amhara	2	63.3 (57.54, 69)	—	—	2	36.7 (33.8, 39.6)
Oromia	2	51.4 (−2.5, 105.2)	3	25 (10.4, 39.6)	3	40 (−25.6, 105.6)
Tigray	3	39.3 (20.3, 58.2)	2	43.7 (40.6, 46.9)	2	47.2 (19.9, 74.5)
SNNP	7	35.7 (21.1, 50.3)	7	47.8 (39.9, 55.6)	7	16.1 (8.4, 23.8)

	# of studies	Inadequate MDD (%) (95% CI)	Adequate MDD (%) (95% CI)
*Study regions*			
Amhara	5	47.2 (29.4, 66)	53 (35.4, 71.3)
Oromia	3	52.6 (24.5, 80.6)	47.5 (19.4, 75.5)
SNNP	2	60.1 (44.4, 75.8)	39.9 (24.2, 55.6)
Others	2	68.9 (18.1, 119.7)	31.1 (−19.7, 82)

### 3.5 Dietary practice and meal frequencies of pregnant and lactating women in Ethiopia

**FIGURE 5:**
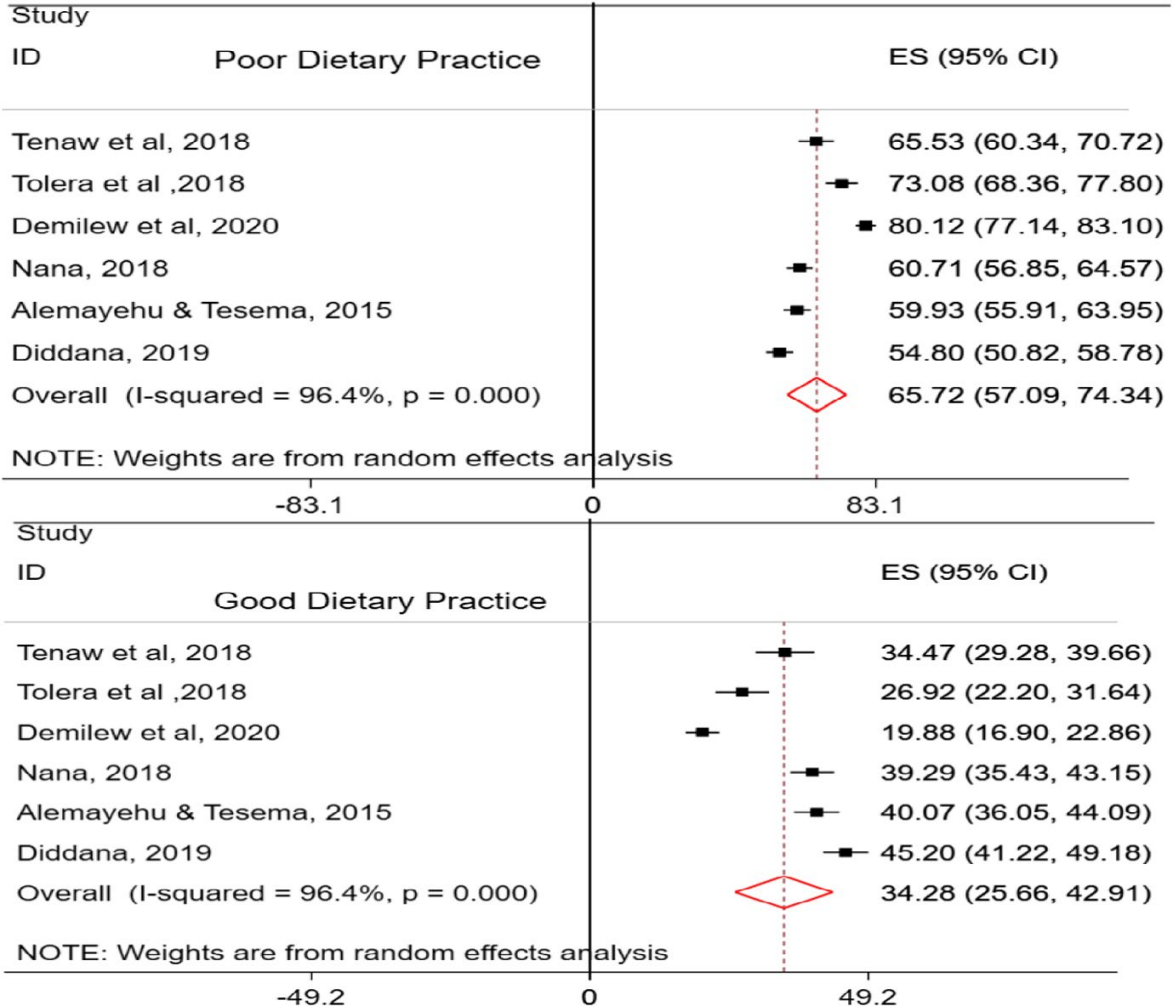
Dietary practices of pregnant women in Ethiopia

The pooled prevalence of dietary practice of pregnant was computed using six studies (Alemayehu & Tesema, 2015; Demilew et al., 2020; Diddana, 2019; Nana & Zema, 2018; Tenaw et al., 2018; Tolera et al., 2018), which were conducted in different regions of Ethiopia. The prevalence of poor dietary practice was found to be 65.72% (95% CI: 57.1, 74.34; *I* ^2^ = 96.4, *p* <.01), and 34.28% (95% CI: 25.66, 42.91; *I* ^2^ = 96.4, *p* ≤ .01) of pregnant women had a good dietary practice (Figure 5).

Regarding the meal frequency of pregnant and lactating women in Ethiopia, it was reported by 23 studies (Abriha et al., 2014; Alemayehu et al., 2015; Alemayehu & Tesema, 2015; Aliwo et al., 2019; Boke & Geremew, 2018; Kobiro et al., 2020; Demilew et al., 2020; Desalegn et al., 2018; Desta et al., 2019; Diddana, 2019; Duko et al., 2018; Engidaw et al., 2019; Eramo, 2018; Gizahewu et al., 2019; Haileslassie et al., 2013; Hailu & Woldemichael, 2019; Jemal & Awol, 2019; Kumera et al., 2015; Nigatu et al., 2018; Sitotaw et al., 2017; Sonko, 2016; Tikuye et al., 2019; Weldehaweria et al., 2016) with a total of 9,447 study subjects. Of the total study subjects, 7,554 (80%) of pregnant and lactating women ate ≤3 times per day, whereas 1854 (20%) of them ate ≥4 times per day (Table 1).

### 3.6 Dietary pattern of pregnant and lactating women in Ethiopia

**FIGURE 6:**
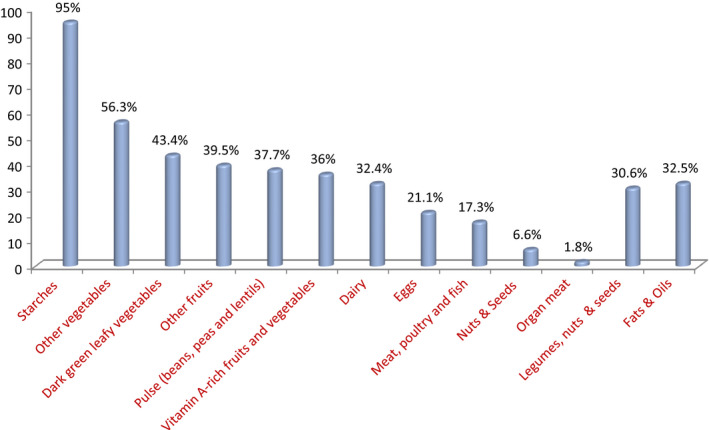
Dietary diversity patterns and food groups consumed by pregnant and lactating women in Ethiopia

In the current study, the food types that were consumed by pregnant and lactating women in Ethiopia were explored and analyzed accordingly. A total of 26 studies were used to compute the pooled quantities of food types. Seven of the studies (Alemayehu et al., 2015; Boke & Geremew, 2018; Duko et al., 2018; Eramo, 2018; Haileslassie et al., 2013; Tikuye et al., 2019; Weldehaweria et al., 2016) were conducted among lactating women, and 19 of them (Ali, 2019; Asayehu et al., 2017; Kobiro et al., 2020; Delil et al., 2018; Demilew et al., 2020; Desalegn et al., 2018; Desta et al., 2019; Diddana, 2019; Endalifer et al., 2019; Hailu & Woldemichael, 2019; Jemal & Awol, 2019; Kumera et al., 2015; Lebso et al., 2017; Nigatu et al., 2018; Samuel et al., 2020; Shenka et al., 2018; Sonko, 2016; Yeneabat et al., 2019; Zerfu & Biadgilign, 2018) were conducted among pregnant women. Out of a total of 9,798 study subjects, the majority (9,310) of them ate starches (grains, white roots and tubers, and plantains). The other food types that were consumed by the study subjects were other vegetables (5,521); dark green leafy vegetables (4,249); other fruits (3,868); pulse (beans, peas, and lentils) (3,696); vitamin A‐rich fruits and vegetables (3,532); dairy (3,171); eggs (2066); meat, poultry, and fish (1695); and nuts and seeds (650). In addition, the other food types other than the 10 food types recommended by FAO (FAO, 2016b) that were consumed by study participants were fats and oils (3,185); legumes, nuts, and seeds (3,002); and organ meat (180) (Figure 6).

## 4 DISCUSSION

In this systematic review and meta‐analysis, the pooled prevalence of dietary diversity scores, minimum dietary diversity, dietary practice, and the food groups of pregnant and lactating women in Ethiopia were computed. The meal frequencies and mean dietary diversity scores of pregnant and lactating women in Ethiopia were explored and computed accordingly.

The pooled prevalence of low DDS of pregnant and lactating women in Ethiopia is 37.1% and 50.31%, respectively. The combined pooled prevalence of low DDS is found to be 42.76% with marked heterogeneity among the included studies. Likewise, the pooled prevalence of medium DDS of both pregnant (41.55%) and lactating women (41.22%) is nearly similar to a combined pooled prevalence of 41.38%. Similarly, more than quarters (28.7%) of pregnant and lactating women are found to have high DDS. Specifically, 39.27% of pregnant women and 9.1% of lactating women have high DDS. The current finding differs from a study finding in Burkina Faso where the low, medium, and high DDS of women were 16.3%, 39.2%, and 44.5%, respectively (Abris et al., 2018). The possible explanation for the dissimilarities could be due to differences in socioeconomic and demographic status of study subjects. The difference in the design of the studies might also be accounted for the variation. The mean DDS of pregnant and lactating women in Ethiopia is 3.99 ± 0.20, which is lower than a mean DDS of women in Burkina Faso (6.5 ± 1.5) (Abris et al., 2018) and Malawi (4.06 ± 1.18) (Walters et al., 2019), but comparable with the findings in Kenya (3.78 ± 0.99) (Gitagia et al., 2019) and Nepal (3.9 ± 1) (Henjum et al., 2015).

The minimum dietary diversity (MDD) of women in Ethiopia is classified as inadequate and adequate. In the current study, 56.7% of pregnant and fifty percent of lactating women have inadequate MDD. The combined pooled prevalence of inadequate MDD of pregnant and lactating women is 54.54%. In contrast, the pooled prevalence of adequate MDD of pregnant and lactating women in Ethiopia is estimated to be 45.9% and 44%, respectively. Adequate MDD women in this study (44.3%) is relatively lower than the findings of the studies in the slums of Kolkata (India) and Saudi Arabia, where adequate MDD of women of reproductive age were 46.2% (Pal et al., 2017) and 54% (Ahmed & Salih, 2019), respectively. But, the current finding is higher than a study finding in Kenya where 19.8% of women of reproductive age had adequate MDD (Gitagia et al., 2019). The present finding is also higher than the MDD of pregnant women (31%) in Malawi (Walters et al., 2019). These variations could be attributed by sociodemographic differences in the study of the subjects. The presence of relatively higher rate of inadequate MDD of women in Ethiopia could be associated with inadequate availability and access to food items both at the household level and at the market level (Ambikapathi et al., 2019). This could be also the cause for high burden of micronutrient deficiency among women in Ethiopia (Haidar, 2010; Harika et al., 2017) like what is happening in the other parts of the world (Gernand et al., 2016; Rahmannia et al., 2019) in that dietary diversity is the main predictor of micronutrient adequacy. Nonetheless, the burden of micronutrient deficiency is significantly higher in resource‐poor settings where micronutrient intake of women is suboptimal (Torheim et al., 2010).

In the present study, the dietary practices of pregnant and lactating women were explored, but all the studies included in this meta‐analysis were studies conducted among pregnant women. In Ethiopia, the majority (67.7%) of pregnant women are found to have poor dietary practice and around one‐third (34.3%) of them have good dietary practices. This implies that poor dietary practice is considerably high. The possible elucidation for this is that most of the pregnant and lactating women in Ethiopia have suboptimal feeding practices. For instance, in this meta‐analysis, most of the women (80%) eat foods less than or equal to three times per day. Suboptimal meal frequency could also predispose women to have low DDS and inadequate MDD. In addition, restriction of taking some of the food items due to food taboos such as avoid taking of linseed, honey, milk, and nuts not to give birth of a “fat baby” and avoiding green vegetables not to give birth to a bald baby could be the cause for poor dietary practice of pregnant women in Ethiopia (Vasilevski & Carolan‐Olah, 2016). The dietary practice of the general population in Ethiopia is also relatively poor (Keflie et al., 2018). This study is also supported by a systematic review, which revealed women in most developing countries had poor dietary practices (Tafese & Kebebu, 2017).

Coming to the food groups consumed by pregnant and lactating women in Ethiopia, starchy foods are the most common staple foods consumed by the majority (95%) of women. This substantiates the monotonic nature of foods consumed by most women in the developing world (Kang et al., 2019; Perumal et al., 2013; Sirotin et al., 2012). Other vegetables are the second (56.3%) main food items consumed by pregnant and lactating women in Ethiopia. Likewise, very limited numbers of pregnant and lactating women consume animal source foods (dairy (32.4%); eggs (21.1%); meat, poultry, and fish (17.3%); and organ meat (1.8%)). The other food types such as fruits and vegetables are consumed below the WHO recommended level like other low‐ and middle‐income countries (Abate et al., 2019). All those findings depicted that the feeding practices of women are suboptimal, and this could be the reason why most Ethiopian women are living with micronutrient deficiencies (Haidar, 2010). This could also be the possible rationale for 22% and 24% of Ethiopian women (15–49 years of age) to be undernourished and anemic, respectively (EDHS, 2016). Organ meat (the richest source of minerals such as iron and others and vitamins such as A, B, D, E, and K) (Ahmad et al., 2018) consumption of women is very limited, which could be the cause for micronutrient deficiencies and anemia in women. In Ethiopia, the prevalence of obesity among women is increasing alarmingly (EDHS, 2016) and this might be attributed by inappropriate feeding practice of women in that considerable number of women eat monotonous food items, mainly starchy foods with high glycemic index. In addition, significant proportions (32.5%) of women also consume unhealthy diets such as saturated fats and trans‐fats, which could lead to obesity.

### 4.1 Strengths and limitations

In this systematic review and meta‐analysis, all efforts were made to retrieve published and unpublished articles from both reputable electronic database and gray literature sources. Accordingly, the pooled estimates of DDS, MDD, and dietary practices of both pregnant and lactating women (the vulnerable population group for malnutrition) were computed. These findings will have an indispensable implication for policymakers, program planners, and healthcare providers and nutritionists to design nutrition intervention programs for pregnant and lactating women, accordingly. The finding will have special importance for the Ministry of Health of Ethiopia to enact in this striking issue. However, the limitations of this study were difficulty in computing the pooled DDS, MDD, and dietary practices for women; variation of outcome measures among included studies; and incompleteness of reports that could compromise the representativeness of pooled estimates.

## 5 CONCLUSION

In conclusion, pregnant and lactating women were food found to have poor dietary diversity and practice with most women eat monotonous food, mainly starchy foods. Likewise, a considerable number of women eat unhealthy diets such as saturated fats and oils. Consumption of animal source foods, especially fish and organ meat, is very limited. It is recommended to the Ministry of Health of Ethiopia, policymakers, and nongovernmental organizations to redesign nutritional intervention programs for pregnant and lactating women. This could decrease the high burden of micronutrient deficiencies of women in the country. Improving the feeding practice and nutritional adequacy of women could also break the intergenerational effect of malnutrition.

## CONFLICT OF INTEREST

The authors declare that they do not have any conflict of interest.

## AUTHOR CONTRIBUTIONS

ZWB, EGA, and AA conceptualized the study. ZWB, TW, and AA involved in formal analysis. ZWB, EGA, AA, and TW designed methodology. ZWB, TW, and AA provided software. ZWB, TW, and AA wrote the original draft. ZWB, EGA, AA, and TW wrote, reviewed, and edited the manuscript. Finally, all authors read and approved this manuscript for publication.

## ETHICAL APPROVAL

It is not applicable for this work.

## Supporting information

Figure S1

## Data Availability

All relevant data are within the manuscript and supporting files.
