# Supplementary material for: Dietary diversity and practice of pregnant and lactating women in Ethiopia: A systematic review and meta‐analysis
Source: Food Sci Nutr. 2021 Mar 16;9(5):2686–702. doi: 10.1002/fsn3.2228 (PMC8116864; doi:10.1002/fsn3.2228)
Supplement: Supplementary file 1 — Figure S1 [file FSN3-9-2686-s001.docx]

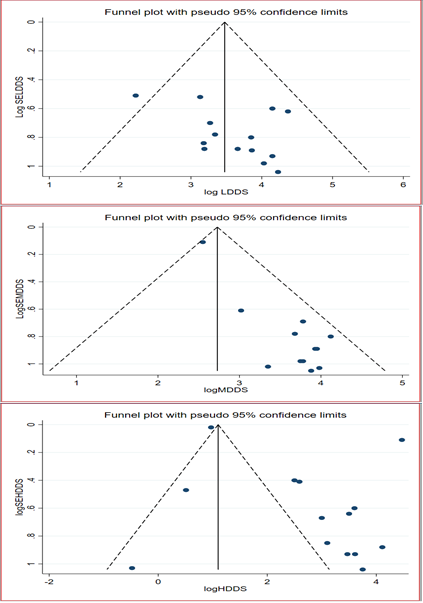


**Supplementary Figure:** Funnel plots of LDDS, MDDS and HDDS of pregnant and lactating women in Ethiopia.
